# Supplementary material for: The Loss of Complex I in Renal Oncocytoma Is Associated with Defective Mitophagy Due to Lysosomal Dysfunction
Source: Int J Mol Sci. 2025 Aug 7;26(15):7654. doi: 10.3390/ijms26157654 (PMC12347756; doi:10.3390/ijms26157654)
Supplement: Supplementary file 1 [file ijms-26-07654-s001.zip › ijms-3687827-supplementary.pdf]

Supplemental materials and methods

Respirometry in frozen samples (RIFS) supplemental methodology

The RIFS protocol allows for assessment of maximal mitochondrial respiratory capacity in previously-frozen biological samples for situations when freshly isolating mitochondria is not feasible. Respiration is measured with substrates for Complex I, II, and IV of the electron transport chain. NADH is used to measure respiration through Complex I. Succinate (Complex II substrate) with rotenone (Complex I inhibitor) is used to measure respiration through Complex II. TMPD and ascorbate (to keep TMPD in the reduced state) is used to measure Complex IV activity. This method reliably measures maximal oxygen consumption rates and allows for side-by-side comparisons while reducing the amount of starting material required. It is important to note that State 3 respiration (ATP synthesis capacity) cannot be measure from frozen samples.

DQ-BSA lysosomal activity assay

YUNK1 cells (previously derived from uninvolved renal cortical tissue14) were labeled with 1 µg/mL DQ Red BSA (Thermo Fisher, D12051) for 6 hours, or labelled with 0.3µM LysoTracker Green (Thermofisher, Cat # L7526) for 30 min, in combination with 1 µg/mL CellTracker Deep Red (Thermo Fisher, C34565) for 30 min and Hoechst (Invitrogen Cat#H3570) for 30 min in staining medium (DMEM no phenol red, Gibco Cat # 21063029, 1% FBS) at 37°C, washed twice, and cells were imaged alive. Images were acquired using a Zeiss LSM 710 confocal microscope and processed with Zen 2011 SP3 software. Lysosome density was calculated as the average intensity of LysoTracker Green above threshold per area of CellTracker Deep Red, lysosomal activity was calculated as the average intensity of DQ Red BSA above threshold per area of CellTracker Deep Red.

Lysosomal Imaging

Lysosome number and activity were assessed by staining cells with XuM LysoTracker Green or XuM BSA-DQ-Red, respectively. Cells were also co-stained with XuM Cell Tracker Deep Red (whole cell) and 1µg/ml Hoechst (nuclei) for further analysis based on cellular mask. Before measurement, the cells were washed twice with medium and phenol-red fresh medium was added. Imaging was performed in triplicates, using confocal mode and Z-stacks of 1 µm (6 µm total) with the ImageXpress Molecular Device system (40x water objective). Excitation and emission filters used for the combination of dyes: Hoechst (ex. 360-400, em.410-480), LysoTracker (ex. 460-490, em. 500-550), BSA-DQ-Red (ex. 560-580, em. 590-640), cell Tracker Deep Red (ex. 630-650, em. 640-680). Analysis was performed with Molecular Device (Harmony 4.1) software by masking whole cells using Cell Tracker and Hoechst staining. Only whole cells were further analyzed to have intensity of DQ-BSA in the mask created by thresholding the Lysotracker channel. Lysotracker mask was used for calculation of morphological parameters.

A

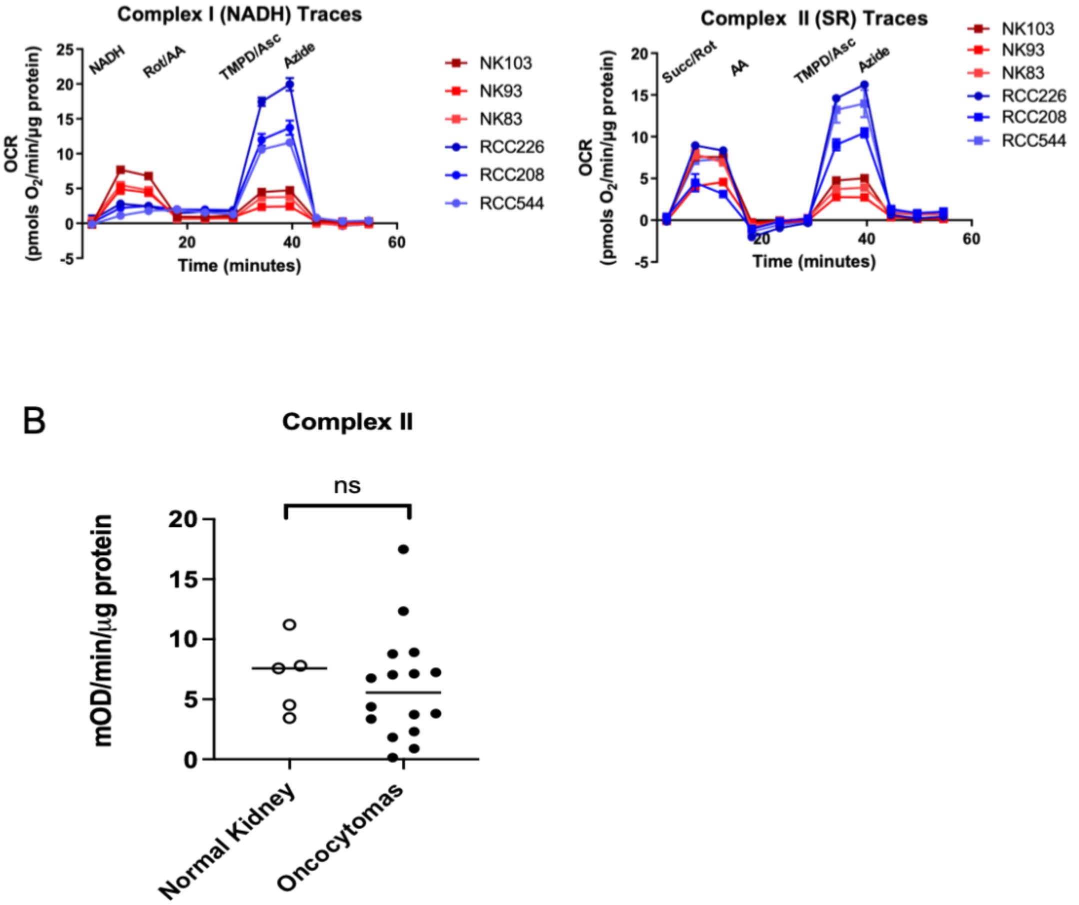

Figure S1: A. OCR tracings from normal kidney and oncocytomas, 3 representative tracings from each group were depicted here. B. Complex II OCR from frozen oncocytoma and normal kidney lysates.

Figure S2

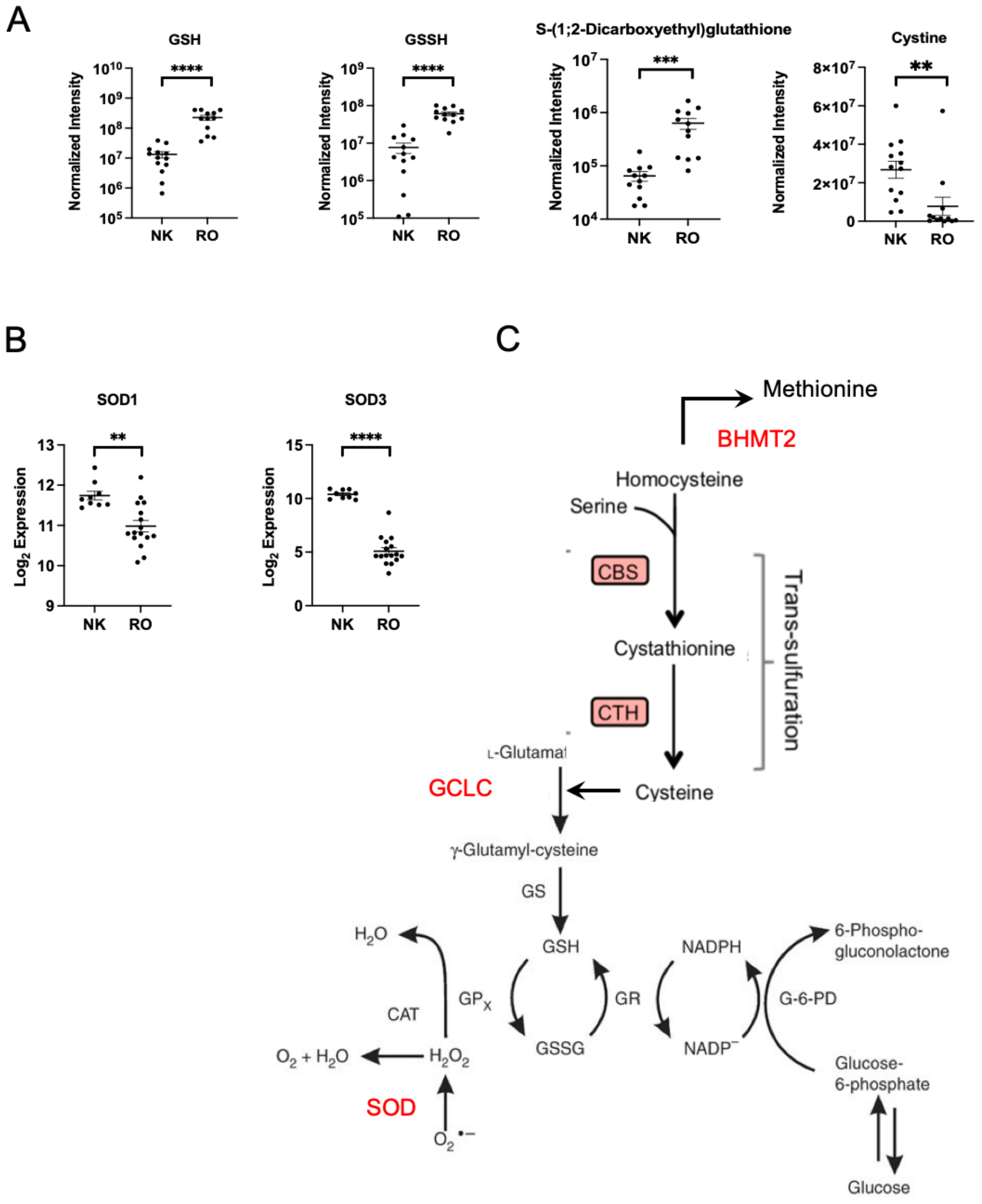

Figure S2: A. Individual metabolite profiling using LC-MS; B. Gene expression of key enzymes in glutathione metabolism pathways using Nanostring nCounter metabolic panel. C, diagram of glutathione metabolism pathway.

Figure S3

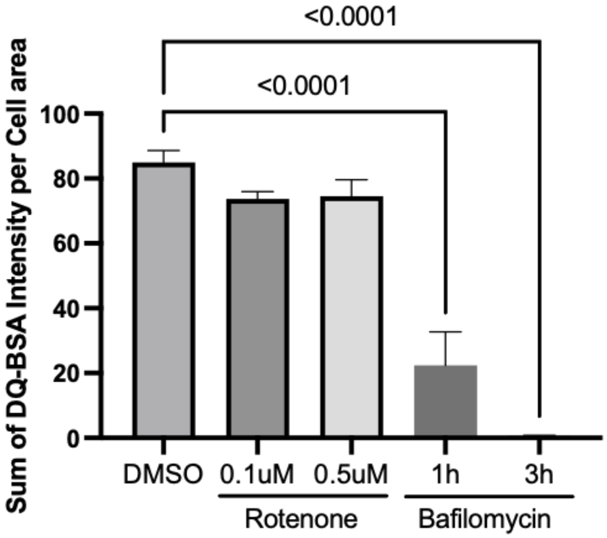

Figure S3: Lysosome acidification function is not altered by rotenone treatment in YUNK1 cells.
